# Mechanism and regulation of P element transposition

**DOI:** 10.1098/rsob.200244

**Published:** 2020-12-23

**Authors:** George E. Ghanim, Donald C. Rio, Felipe Karam Teixeira

**Affiliations:** 1 Department of Molecular and Cell Biology, University of California, Berkeley, CA 94720, USA; 2 California Institute for Quantitative Biosciences, University of California, Berkeley, CA 94720, USA; 3 Department of Genetics, University of Cambridge, Cambridge CB2 3EH, UK

**Keywords:** P transposable elements, *Drosophila*, hybrid dysgenesis, THAP9

## Abstract

P elements were first discovered in the fruit fly *Drosophila melanogaster* as the causative agents of a syndrome of aberrant genetic traits called hybrid dysgenesis. This occurs when P element-carrying males mate with females that lack P elements and results in progeny displaying sterility, mutations and chromosomal rearrangements. Since then numerous genetic, developmental, biochemical and structural studies have culminated in a deep understanding of P element transposition: from the cellular regulation and repression of transposition to the mechanistic details of the transposase nucleoprotein complex. Recent studies have revealed how piwi-interacting small RNA pathways can act to control splicing of the P element pre-mRNA to modulate transposase production in the germline. A recent cryo-electron microscopy structure of the P element transpososome reveals an unusual DNA architecture at the transposon termini and shows that the bound GTP cofactor functions to position the transposon ends within the transposase active site. Genome sequencing efforts have shown that there are P element transposase-homologous genes (called THAP9) in other animal genomes, including humans. This review highlights recent and previous studies, which together have led to new insights, and surveys our current understanding of the biology, biochemistry, mechanism and regulation of P element transposition.

## 1. Hybrid dysgenesis, horizontal gene transfer and a natural gene drive

**Figure 1.**
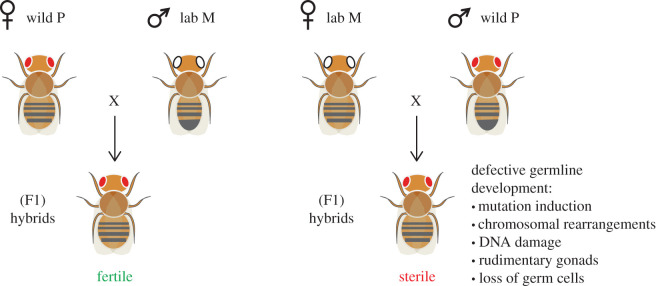
Genetics of hybrid dysgenesis. The reciprocal crosses of hybrid dysgenesis are indicated. The dysgenic cross involves mating wild P strain males to lab M strain females resulting in dysgenic ovaries. This results in high levels of P element transposition, induction of mutations, chromosomal breaks and rearrangements and germ cell death. By contrast, when lab M strain males are mated to wild P strain females, normal ovaries are produced because the P strain females have the repressive state known as ‘P cytotype’. Adapted from [3].

P elements were discovered in the mid-1970s by population geneticists when wild *Drosophila melanogaster* strains were brought into captivity and mated to laboratory strains that had been in captivity since the early 1900s [1,2]. Surprisingly, when male flies from wild strains (termed P or paternally contributing) were mated to female flies from laboratory strains (termed M or maternally contributing) a number of abnormalities were observed, including sterility due to rudimentary gonad development, high rates of mutation and chromosomal rearrangements. By contrast, the resulting progeny from the reciprocal cross (lab male (M) by wild female (P)) were normal and fertile (figure 1). Collectively, this syndrome of traits was termed hybrid dysgenesis [4–6].

It is believed that P elements were introduced into *D. melanogaster* from another *Drosophila* species by horizontal gene transfer via a parasitic mite, early in the part of the twentieth century [7,8]. Because the progeny of dysgenic crosses are sterile [5,6], there is a strong biological selection for the ability to repress P element mobility in the wild. Despite this, P elements spread throughout wild populations in about 30 years. In fact, all *D. melanogaster* isolated from the wild since the 1980s have P elements [9]. This invasion constitutes a natural gene drive [10–12] and population analyses indicate that a similar invasion is currently under way in *D. simulans* wild populations [13–15].

The first clue that hybrid dysgenesis was caused by transposable elements came from the fact that some of the resulting mutations were notably unstable and could revert [2,16]. As a direct test of this idea, a series of dysgenic crosses were carried out with the goal of isolating eye colour mutants, since the *Drosophila white* locus had been cloned and was a good target for identifying the nature of hybrid dysgenesis-induced mutations [17,18]. This analysis led to the identification of DNA insertions into the *white* locus and the reversion of these mutations resulted in the loss of those DNA elements. These DNA insertions were subsequently molecularly isolated and termed P elements [18,19].

## 2. Maternal effect of P cytotype and the involvement of the PIWI-interacting RNA pathway

**Figure 2.**
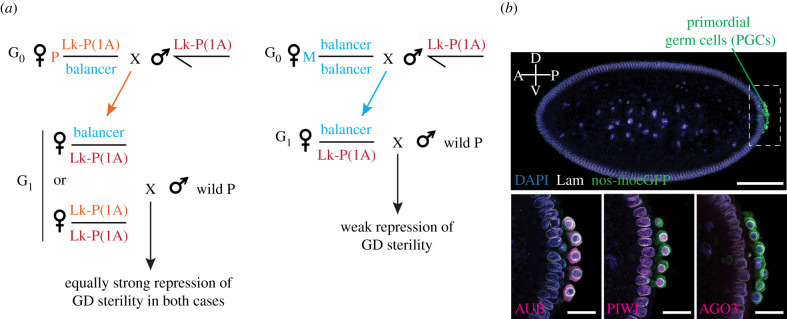
Maternal effect genetics of P cytotype during hybrid dysgenesis and the maternally inheritance of PIWI proteins. (*a*) The repressive P strain, Lk-P(1A), can transmit P cytotype across multiple generations in a maternal effect pattern, independently of the inheritance of the maternally derived Lk-P(1A) chromosome. That is (left) if mothers (G_0_) carry Lk-P(1A), then the G_1_ females carrying either the maternally inherited or the paternally inherited Lk-P(1A) can propagate the ability to repress hybrid dysgenic sterility to the next generation. In the reciprocal test cross (right), females not carrying Lk-P(1A) are mated to Lk-P(1A) males, and the G_1_ progeny females carrying the paternally inherited Lk-P(1A) chromosome but lacking the maternally deposited Lk-P(1A)-derived cytotype cannot provide G_2_ females with strong repression of GD sterility [21]. The idea is that the oocytes from the Lk-P(1A) grandmothers deposit maternal components that create P cytotype. This is due to transmission of piRNAs in the oocytes derived from Lk-P(1A) mothers. (*b*) Confocal images of Drosophila embryos expressing the germline marker nos-moeGFP [22], approximately 1.5–2 h after egg laying—during primordial germ cells (PGCs) formation at the posterior pole of the embryo and before zygotic genome activation. Bottom images show the incorporation of the maternally inherited AUB (cytoplasmic) and PIWI (nuclear) proteins—but not AGO3—into the budding PGCs. Embryos were stained for GFP (germ cells, green); DAPI (DNA, blue), Lamin Dm0 (nuclear envelope, white) and PIWI family proteins (magenta) AUB (bottom left), PIWI (bottom centre) and AGO3 (bottom right). Scale bars: 100 µm (top) and 20 µm (bottom).

The dissection of the maternal effect observed on reciprocal crosses between *D. melanogaster* wild and lab strains ultimately led to the description of one of the best-characterized examples of transgenerational epigenetic inheritance in animals. Despite initial difficulties in identifying the molecular basis of the factor conferring transgenerational protection, a series of elegant genetic analyses were used to definitively demonstrate that the epigenetic information was provided by determinants found in the oocyte cytoplasm rather than the maternal DNA itself [17–20]. One of the clearest examples was provided by the genetic experiments relying on the naturally occurring and genetically traceable P element insertion Lk-P1A, which is located within the telomeric associated sequence on the X-chromosome (X-TAS) and that is individually able to suppress dysgenesis (figure 2*a*) [20,21,23]. Such experiments provided genetic proof that the transgenerational protection relied on the presence of the maternally provided Lk-P1A-derived cytoplasm rather than the maternally provided Lk-P1A chromosome [21,24–26]. From there, a series of genetic, genomic, biochemical and developmental studies culminated with the identification of an evolutionarily conserved small RNA interference (RNAi) pathway in animals [27,28]. These studies revealed that the transgenerational epigenetic information is provided by maternally deposited piwi-interacting small RNAs (piRNAs) [29,30] and relies on the function of the respective piRNA biogenesis pathway [31–33].

piRNAs are small RNA molecules (23–29 nt in *Drosophila*) generated through the processing of larger RNA transcripts and are eventually loaded into Argonaute effector protein complexes of the PIWI family (Piwi, Aub and Ago3 in Drosophila; reviewed in [34]). Similar to other RNAi systems, piRNAs endow PIWI proteins with sequence-specificity by complementary Watson–Crick base pairing with their targets, which are mostly derived from transposable elements and other genomic repeats [28,32]. In flies and in mammals, the expression of piRNAs, as well as of the PIWI proteins, is mostly restricted to the gonads, and Aub and Piwi had been initially identified for their role in germline specification and development rather than their role in the piRNA pathway [35–38]. Most importantly, from a transgenerational perspective, Aub and Piwi proteins—likely loaded with piRNAs produced during oogenesis—are maternally deposited at the posterior pole of the oocyte in a specialized cytoplasm known as the germ plasm, which is then incorporated into the developing germ cells during embryogenesis in the next generation (figure 2*b*) [30,37,39]. This process is thought to account for the epigenetic nature of the P cytotype, through a series of genetic and molecular studies revealing that maternally provided piRNAs are sufficient to trigger homology-dependent RNAi silencing *in trans*, promote chromatin changes at target loci, regulate P element expression, and kick-start piRNA production in the germline of the next generation [23,26,30,33,40–42].

Over the last 40 years, the study of the P cytotype protection has provided the paradigm of epigenetic transgenerational phenomena that culminated with the description of piRNAs as the source of epigenetically inherited information [25,29,30]. While much progress has been made, some aspects of the transgenerational model have not yet been directly dissected, largely due to technical and developmental challenges. Notably, the fact that PIWI proteins are required for germline development and germ plasm assembly has imposed constrains to experimental design [34–38]. In this context, it will require the development of new strategies and methods to approach some of these key questions, including the molecular mechanisms by which transgenerationally inherited piRNAs act on their targets in developing germ cells and how maternally provided PIWI-piRNA complexes perpetuate their production through multiple generations.

## 3. Temperature sensitivity and the development of dysgenic germ cells

An intriguing feature of hybrid dysgenesis that readily captivated the attention of researchers was its temperature-sensitivity [4]. This feature was characterized early on by Kidwell & Novy [5] and by Engels & Preston [43]: the narrow temperature range between 24 and 26°C defines a dramatic threshold for change in phenotype. F1 hybrid progeny raised at temperatures below this range show no clear dysgenic phenotype, while sterility is observed in individuals kept in temperatures at or higher than 27°C; with female sterility tending to exceed male sterility. For individuals grown at 25°C, variable gonadal dystrophy penetrance is observed, with a fraction of individuals maintaining low fertility, which can be progressively but modestly improved in an age-dependent manner [12]. While the basis of the temperature sensitivity is not currently known, *in vitro* biochemical analyses revealed that P element transposase is less active at lower temperatures [44].

**Figure 3.**
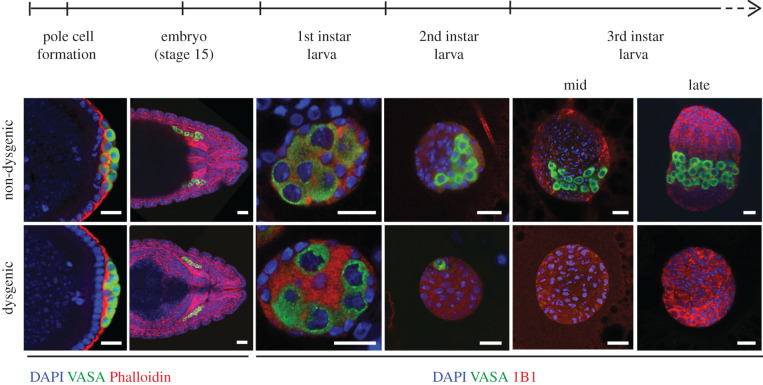
Fate of dysgenic germ cells. Confocal images of germ cell development in non-dysgenic and dysgenic progeny during embryonic and larval stages. Embryos and larvae were stained for Vasa (germ cells, green); DAPI (DNA, blue); phalloidin (F-actin, red; embryo stages); 1B1 (somatic cells and spectrosomes, red; larval stages). Pole cell formation is approximately 1.5 h after egg laying (AEL); embryo stage 15 is 10–12 h AEL; first instar larva is 22–48 h AEL; second instar larva is 48–72 h AEL; third instar larva is 72–120 h AEL. Scale bars: 20 µm. Adapted from [33].

The characterization of the developmental defects associated with hybrid dysgenesis, which was initially restricted to macroscopic analyses [43,45], revealed that dysgenic individuals present rudimentary adult gonads characterized by near-complete to complete loss of germ cells. Microscopy analysis carried out with progeny raised at restrictive temperatures (29°C) revealed that dysgenic germ cells have a relatively normal embryonic development, with primordial germ cells (PGCs) being formed in the posterior pole of the embryo at similar numbers in comparison to non-dysgenic progeny [33,46]. Mid- and late-embryonic development, encompassing germ cell migration and gonad coalescence, are also mostly unaltered in dysgenic progeny. During larval stages however, dysgenic germ cells sharply decrease in number, with female larvae being completely devoid of germ cells by late-larval development (figure 3) [33,46]. Developmental analyses corroborated results obtained early on from temperature shift experiments, which narrowed the temperature-sensitive window leading to complete sterility to the period between 10 h after egg laying and 4 days of development—a period that spans from mid-embryogenesis until late-larval development [5,43]. At one end, the delayed beginning of the temperature-sensitive window may reflect the fact that PGCs are transcriptionally quiescent until mid-embryogenesis, with the zygotic genome being activated while germ cells are reaching the somatic gonad [47]. At the other end, it is likely that the germ cell fate transition from PGCs to germline stem cells (GSCs), which occurs during late-larval stages when the somatic niche is formed and becomes active, delineates the end of the temperature-sensitive window that leads to complete sterility. In agreement with the idea of adult GSCs being less sensitive to dysgenesis than embryonic PGCs, female progeny raised at the permissive temperature (18°C) and shifted to restrictive temperatures (29°C) during adulthood do not become fully sterile. Instead, following a temporary halt in GSC differentiation, fertility is restored to wild-type levels a few days after the beginning of the exposure to restrictive temperatures [5,48]. In this case, as well as what was observed in individuals grown at 25°C, halt in germline development in response to dysgenesis involves the activity of the chk2 DNA damage checkpoint kinase [48,49].

## 4. Organization and molecular biology of P elements

**Figure 4.**
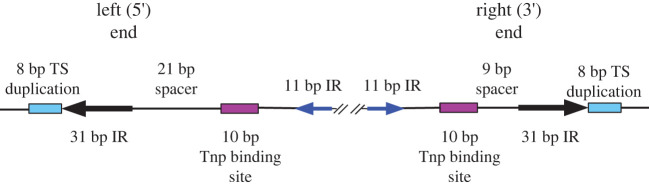
Sequence features at the P element termini. P elements have 31 bp terminal inverted repeats, internal site-specific binding sites for the transposase protein (TNP) and 11 bp internal inverted repeats. There are 8 bp direct target site duplications flanking the P element insertions. Finally, note the distinct spacing between the 31 bp inverted repeats and transposase binding sites at the 5′ and 3′ transposon ends. Adapted from [50].

Molecular biology analysis of P elements isolated from P strain genomic DNA libraries showed that there were two types of P elements: full-length 2.9 kb elements and smaller, non-autonomous internally deleted elements ranging from 0.5–2.8 kb [19,50]. The 2.9 kb full-length P element DNA possesses 31 bp perfect terminal inverted repeats, 10 bp internal transposase binding sites and internal 11 bp subterminal inverted repeats (figure 4) [50]. The left 5′ and right 3′ ends differ in the spacing between the terminal inverted repeat and the 10 bp transposase binding site, 9 bp and 21 bp, respectively. This spacing is reminiscent of the 12 and 23 bp recombination signal sequences (RSS) that is recognized by V(D)J recombinases during the somatic DNA rearrangements resulting in the maturation of the immunoglobin genes in developing lymphocytes in vertebrates [51–53]. Moreover, a recent structural study proposed that the P element transposase pairs the target DNA by an induced asymmetry mechanism [53] that is analogous to that observed for V(D)J recombinases [54–56].

**Figure 5.**
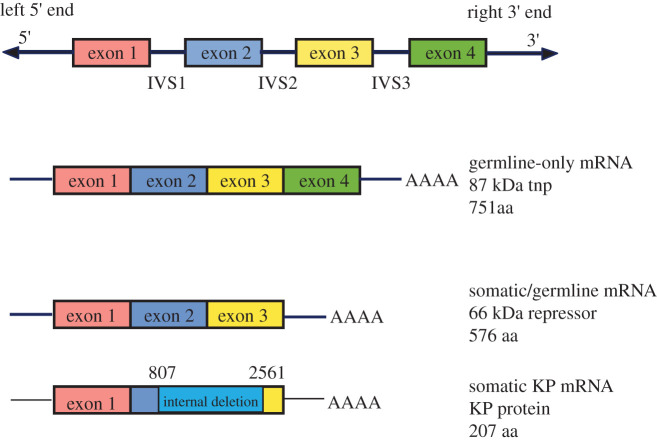
P element open reading frames and derived proteins. Top: complete 2.9 kb P elements encode four protein-coding open reading frames. These can generate two protein-coding mRNAs via alternative splicing with two constitutive introns and one alternative splicing event of intron 3 or IVS3. These mRNAs encode a 66 kDa protein that acts a repressor and an 87 kDa protein that is the active transposase (TNP). Bottom: in the case of small, non-autonomous P elements, they can often encode repressor proteins, such as the 207aa KP protein.

DNA sequencing of the full-length P element indicated that there were four protein-coding open reading frames (ORF 0, 1, 2 and 3; figure 5). Biochemical and molecular biological experiments showed that these ORF could be linked by alternative RNA splicing to encode two proteins: the 87 kDa active transposase protein (TNP) and a shorter 66 kDa protein produced by an intron retention event of the third intron (intervening sequence 3, IVS3) [57–59]. The 87 kDa transposase protein is a complex multi-domain protein that catalyzes the excision and integration of P element DNA [44,50]. Retention of the third intron in somatic cells and in the germline produces an mRNA encoding the 66 kDa protein that acts as a transpositional repressor [60,61]. Importantly, alternative pre-mRNA splicing of the third intron specifically in dysgenic germ cells leads to the production of full-length P element mRNAs encoding for the 87 kDa transposase protein. This restricts the deleterious effects of rampant P element transposition and hybrid dysgenesis to germline cells of dysgenic progeny [59]. This finding was one of the first bona fide examples of functional tissue-specific alternative RNA pre-mRNA splicing.

## 5. P element transposase: domain organization and transposition mechanism

**Figure 6.**
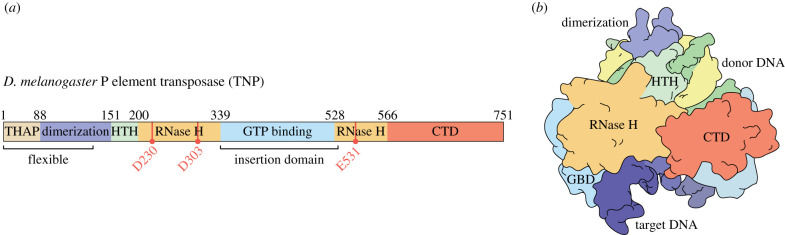
Domain organization of P element transposase. (*a*) Schematic depiction of the domain architecture of P element transposase. Domain boundaries are indicated by amino acid numbers. THAP, THAP DNA-binding domain (beige); dimerization, leucine zipper-dimerization domain (lavender); HTH, helix-turn-helix DNA-binding domain (light green); RNase H, split RNase H domain (dark yellow); GTP binding, GTP-binding insertion domain (GBD, light blue); CTD, C-terminal domain (red). The three catalytic residues in the RNase H domain are indicated by red lines and dots. (*b*) Cartoon of the three-dimensional domain organization deduced from a recent cryo-EM structure, with four of the six domains visible. Coloured as in (*a*), with domains indicated. Donor DNA, P element DNA termini (green and yellow); target DNA (purple). Adapted from [53].

Like other autonomous DNA-based transposons, P elements encode an enzyme, a transposase that is responsible for mobilizing the P element DNA. The P element transposase protein is a complex multi-domain DNA-binding protein, now understood to contain six domains: an N-terminal THAP DNA-binding domain, an adjacent coiled coil, a helix-turn-helix (HTH) DNA-binding domain, an RNase H domain containing a guanosine triphosphate (GTP)-binding domain insertion and an acidic C-terminal domain (CTD; figure 6). This information comes from sequence comparisons, structural and functional studies [62–67].

**Figure 7.**
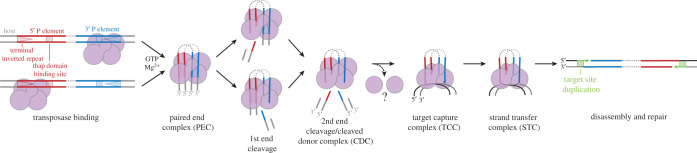
Model of P element transposition pathway. P element transposase first interacts with one P element end via the THAP domain (transposase binding). In the presence of GTP and Mg^2+^, the transposase can form a synaptic or paired-end complex (PEC). The transposon ends are then cleaved asynchronously, releasing the host genomic DNA (cleaved donor complex or CDC). The complex then captures an appropriate target DNA (target capture complex or TCC). Attachment of the transposon ends to the target DNA yields a strand transfer complex or STC, a step which requires GTP and Mg^2+^. Following disassembly and DNA repair, the new insertion site contains 8 bp direct target site duplications flanking the P element. The transposase may undergo an oligomeric rearrangement to account for the tetramer observed at the early stages of transposition by atomic force microscopy studies, and the dimer observed at the final stages by cryo-EM.

Much effort has gone into uncovering the mechanism of P element transposition through the biochemical characterization of the 87 kDa transposase protein. Like other cut-and-paste DNA transposases, P element transposition proceeds in a defined, stepwise manner to ensure accurate DNA cleavage and joining during transposition (figure 7). While the finer details of transposition are specific to each DNA transposon family, the process is generally described by six fundamental steps: transposase-transposon DNA binding, pairing of the transposon ends (synaptic complex formation), donor DNA cleavage, target DNA capture, strand transfer (integration) and disassembly/DNA repair [68–70].

As is the case for other DNA transposons, the P element transposase first assembles with sites on the transposon. Purification and characterization of the transposase protein from *Drosophila* S2 cell nuclear extracts showed that the transposase binds to internal 10 bp sites found at each end of the transposon [71]. Following the initial recognition of a single transposon end, P element transposase captures and pairs the second end in a GTP-dependent manner to form the paired-end complex (PEC; figure 7) [65]. Reconstitution of *in vitro* transposition reactions demonstrated that guanosine triphosphate (GTP) was a required cofactor [44]. Atomic force microscopy volume measurements suggest that a tetrameric form of transposase may be involved in initial synaptic complex assembly [65]. Sequential cleavage at each P element end liberates the transposon from the flanking host genome forming the cleaved donor complex (CDC; figure 7) [65,66]. Like other DNA cut-and-paste transposable elements, DNA cleavage occurs at the 3′ end of the transposon, but on the other strand, 5′ DNA cleavage occurs 17 bp within the P element 31 bp inverted repeats, generating unusual and atypically long 17 nucleotide 3′-single-stranded extensions at the transposon termini (figure 7) [52]. The order and mechanism of strand cleavage are not currently known.

After donor DNA cleavage, the excised transposon-transposase nucleoprotein complex will capture a target DNA (target capture complex; figure 7) and integrate P element (strand transfer complex (STC); figure 7). The sites of transposition are separated by 8 bp, which gives rise to the 8 bp target site duplications (TSDs), after transpososome disassembly and DNA repair. Although P element transposition is not site-specific, a target sequence consensus motif was derived from over 23 000 accurately mapped P element insertions from the *Drosophila* genome project [72]. Transposition preferentially occurs into nearby target sites on the same chromosome (approx. 50–150 kb away) in a phenomenon termed ‘local hopping’ [73]. In addition, P element insertions are highly prevalent at regions around gene promoters and at regions overlapping origins of DNA replication [74,75], which may indicate that the P element transposase has a target preference for regions with an open chromatin topology. The disassembly and DNA repair mechanisms at the target site have not been investigated, but it is understood that the double-strand break generated at the donor site can be repaired through both the homologous recombination-dependent (HR) or non-homologous end joining (NHEJ) DNA repair pathways involving IRBP18/Xrp1, Ku70/80 and the *Drosophila* Bloom's syndrome helicase homologue [76–79].

## 6. Insights from the structure of the P element transposase STC

**Figure 8.**
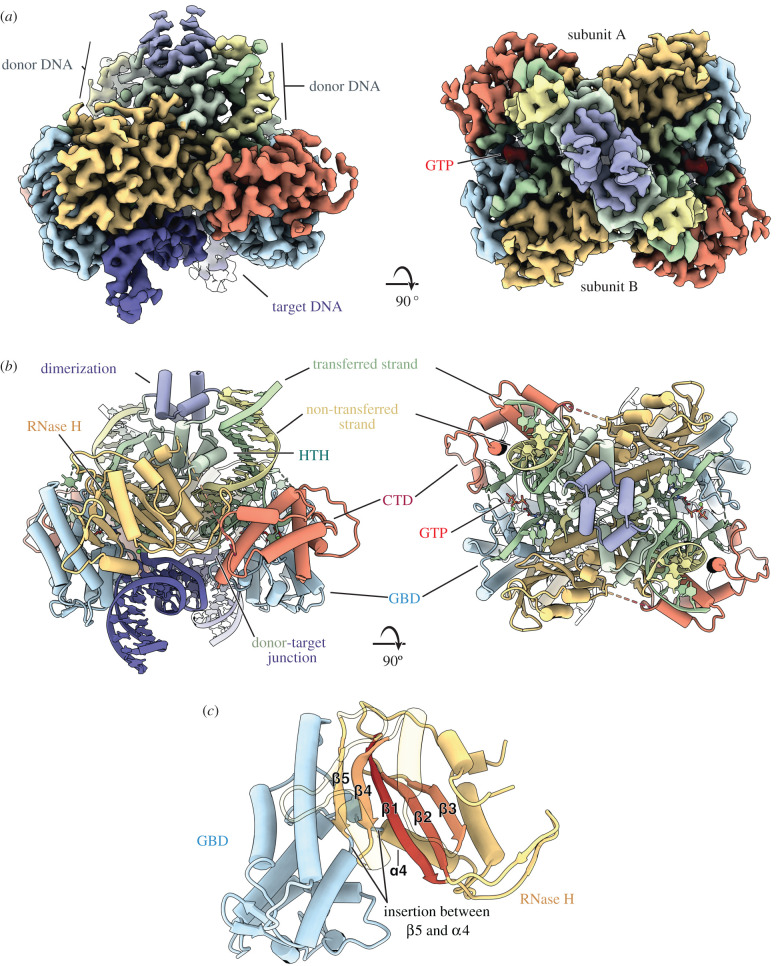
Structure of the P element transposase strand transfer complex (STC). (*a*) Side and top views of the cryo-EM reconstruction at 3.6 Å (left and right, respectively). Domains are coloured as in figure 6 and GTP density is coloured red. Each subunit of the dimer is indicated (right). (*b*) Side and top views of the STC model (coloured as in A, left and right, respectively). Unmodelled connections are shown in dashed lines (dashed green, dashed red). Target DNA is shown in purple, the donor transferred strand in light green and the donor non-transferred strand in yellow. Adapted from [53]. (*c*) GBD insertion into the RNase H fold. Relevant structural elements of the RNase H fold are labelled. The 5 central beta strands are coloured from red to yellow, the overall RNase H domain in yellow and GTP-binding domain in blue (GDB). Insertion position between β5 and α4 is indicated.

Several mechanistic features distinguish P element transposition from the other characterized ‘cut-and-paste’ DNA transposons. Namely, the requirement of the GTP cofactor for the pairing, donor cleavage and strand transfer reactions [44,65,80], and the unusually long 17 nt staggered cleavage at each P element end [52]. To understand the mechanisms underlying the unique features of the P element transposase superfamily, protein-DNA transposition complexes assembled *in vitro* were used for cryo-electron microscopy (cryo-EM) that allowed determination of the structure of the P element transposase STC at 3.6 Å resolution [53]. This post-transposition product complex contains transposase and cleaved P element ends covalently joined to the target DNA. The reconstruction revealed that P element transposase can be divided into six structural domains: the N-terminal THAP DNA-binding domain, a leucine zipper-dimerization domain, a helix-turn-helix DNA-binding domain (HTH), a split catalytic RNase H domain interrupted by a GTP-binding insertion domain (GBD) and lastly a carboxy-terminal domain (CTD; figure 8). Although the THAP domain and the majority of the dimerization domain were not resolved in the reconstruction (presumably due to flexibility), their ability to move provides a rationale for how pairing of the 5′ and 3′ P element ends each with distinct spacing of the inverted repeats and transposase binding sites might occur during initial transpososome assembly [53].

The helix-turn-helix motif constitutes a major structural element capable of binding DNA. The helix-turn-helix domain of P element transposase makes contacts with the inner half of the 31 bp TIRs, engaging through a loop in the minor groove and an HTH α-helix inserted into the major groove (figure 8) [53].

Like other ‘cut-and-paste’ DNA transposases, the catalytic domain of P element transposase adopts a canonical RNase H-like fold, in which a central 5-stranded β-sheet is buttressed above and below by α-helixes [53]. The RNase H-like domain organizes three acidic amino acid residues (D230, D303 and E531), together responsible for the coordination of two divalent metals and for catalyzing the nucleophilic cleavage and joining of DNA phosphodiester bonds [53]. Within the P element STC structure, the RNase H domain is positioned over the target-transposon DNA junction.

Of particular interest in the transposase, RNase H fold is the region connecting *β*5 and *α*4. In some transposases and related retroviral integrases, the *β*5 and *α*4 structural elements are connected by a short, often disordered, loop [81,82]. By contrast, P element transposase has an entire GTP-binding domain inserted between β5 and α4 (figure 8*c*). Domains found at this position are called ‘insertion domains’ and are present in other transposases and transposase-related proteins, such as Hermes, Tn5 and RAG1 [83,84]. Despite structural similarities observed among the insertion domains of all characterized DNA transposases, the one found within P element transposase is the only known GTP-binding insertion domain [53]. Furthermore, P element transposase is unique among the transposase/integrase superfamily in binding GTP as a cofactor for both the cleavage and integration steps of transposition [50].

**Figure 9.**
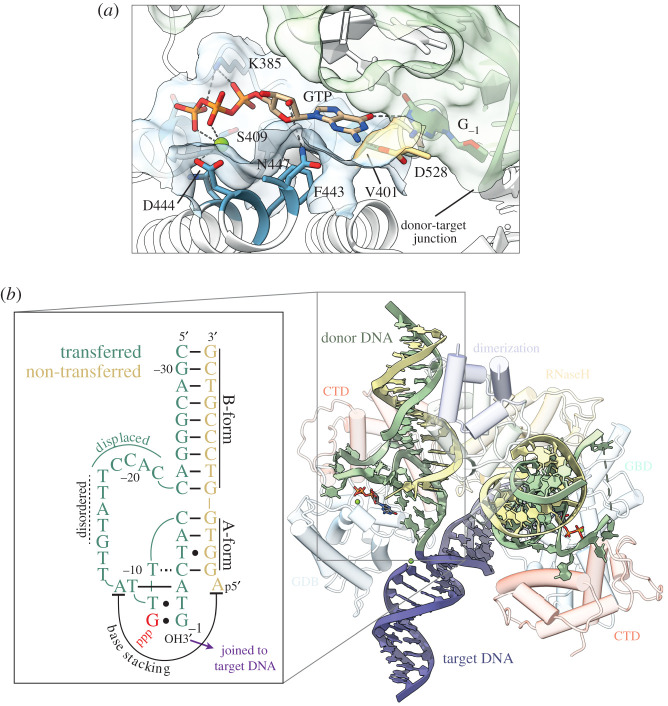
Interactions of the GTP cofactor and unusual structure of the transposon termini in the P element strand transfer complex. (*a*) Close-up of the interaction between GTP, the GBD residues (light blue) and donor DNA (G_-1_, light green). Inferred hydrogen-bonding and electrostatic interactions are shown as grey dashed lines. (*b*) Inset: Schematic of the donor DNA structure. GTP is in red lettering. Watson–Crick base pairings are indicated by solid lines. Non-canonical base pairings are indicated by dots or dotted lines. Nucleotides of the transferred strand are numbered −1 to −31, starting at the 3′ terminal guanosine. Main: structure of the donor DNA within the STC. The transposase protein is faded out for clarity, with relevant domains labelled. The opposing RNase H domain was omitted for clarity. The disordered nucleotides of the transferred strand (−14 to −18) are marked by a dashed green line. Adapted from [53].

The GTP-binding insertion domain (GBD) packs against the RNase H domain and the target-transposon DNA junction within the P element STC structure (figure 8) [53]. The GBD is almost entirely α-helical and is unlike other GTPases, such as ras, dynamin or EF-Tu. A bound GTP cofactor is observed within the GBD and interacts via hydrogen bonding with the terminal base of the transposon DNA, apparently to position the P element DNA for catalysis (figure 9*a*). The mode of GTP binding appears to be unique and is mediated by several residues conserved within members of the P element family (D528, K385, K400, V401, S409, F443, D444 and N447) [53,84]. There is no biochemical evidence for GTP hydrolysis during the cleavage and strand transfer steps of transposition [44,65,66]. Accordingly, residues that could support GTP hydrolysis are either absent or are too far away from the GTP. In addition to binding GTP, the GBD makes numerous contacts with the transposon and target DNAs [53].

The CTD is connected to the RNase H domain by a flexible and disordered linker. The CTD is entirely α-helical and is positioned between the RNase H domain and the GBD of another subunit (figure 8) [53]. Like the GBD, the CTD makes numerous contacts with the transposon and target DNAs. The single-stranded region of the transposon DNA loops out and across the CTD and GBD to make numerous protein-phosphate, and aromatic base stacking interactions all along this path. The CTD extends a basic helix towards the centre of the target DNA. Although the final 17 amino acid residues of the CTD could not be confidently modelled, the region contains many basic residues and is ideally positioned to interact with the target DNA (figure 8) [53].

Two unanticipated observations arose from the P element STC structure, both regarding the transposon DNA. First is the unusual configuration of the transposon DNAs, which adopts a structure more akin to RNA than DNA. A large region of the transferred strand loops out at the CTD, travelling along the C-terminal and GTP-binding domains, then doubles back to base pair with the 5′ portion of non-transferred strand (figure 9*b*) [53]. This base-paired region adopts an A-form helical geometry, typical of RNA–RNA helices, and is required for transposition as supported by mutational analysis [53]. The second unanticipated observation is that the terminal nucleotide appears to interact with the bound GTP cofactor, apparently to position the transposon DNA for catalysis (see above). How the P element DNAs arrive at their observed arrangement within the STC is unclear and undoubtedly involves large conformation changes of both the DNA and protein. Although the order and mechanism of strand cleavage are not currently known, it is possible that like in V(D)J recombination [85,86], there are DNA distortions at the initial donor DNA cleavage stage that lead to the unusual DNA structure found in the STC. The P element STC structure shows GTP positioned up against the transposon DNA near the transposon-target DNA junction, within hydrogen-bonding distance of the terminal transposon DNA nucleotide (between GTP C6 carbonyl and G_-1_ N1). Biochemical experiments with purine nucleoside triphosphate analogues demonstrate that the purine C6 carbonyl group is critical for strand transfer activity [53]. The interaction with GTP appears to alter the trajectory of the transposon DNA strand and positions the attacking 3′OH in the active site, explaining why GTP is required for strand transfer. To our knowledge, this role for GTP has not been observed in other known transposase nucleoprotein complexes that have been studied.

The P element transposase STC structure provided the first view of the P element superfamily of eukaryotic transposases. The unusual nature and high resolution of the structure offered new insights into P element transposition and indicates a transposition pathway mechanistically and fundamentally distinct from other known cut-and-paste DNA transposases.

## 7. THAP DNA-binding domains and the THAP9 genes in vertebrates

**Figure 10.**
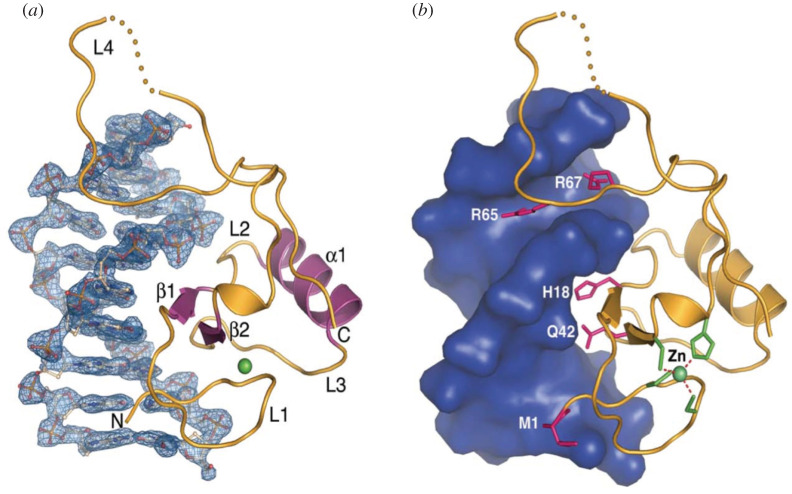
Structure of the P element N-terminal THAP DNA-binding domain complexed with the 3′ P element end binding site. (*a*) THAP domain indicated as a ribbon diagram with the DNA shown in detail. Note the two β-strands in the major groove and C-terminal basic loop in the adjacent minor groove. (*b*) THAP domain indicated as a ribbon diagram with the DNA shown as a space-filling model. Adapted from [67].

With the accumulation of sequenced animal genomes, it was noted that many vertebrate genes shared homology with the N-terminal region of the P element transposase protein [87]. The human genome contains 12 such genes (THAP0-11) that share this N-terminal homology, termed the THAP domain, with human THAP1 the first to be identified. Previous studies of the P element transposase protein had defined the N-terminal region as a C_2_CH zinc-coordinating DNA-binding domain that binds specifically to internal sites adjacent to the 31 bp terminal inverted repeats to tether transposase to the P element DNA during the initial stages of transposition [62]. Subsequent studies using X-ray crystallography showed that the *Drosophila* P element THAP domain bound to the P element DNA using a bipartite major–minor groove mode, unlike other site-specific DNA-binding proteins (figure 10) [67]. Although prevalent, THAP domains are restricted to animal genomes and are not found in plants, fungi or bacteria.

In the human, and other animal genomes, there are genes that not only have an N-terminal THAP domain but have homology along the entire length of the gene to *Drosophila* P element transposase. These genes are designated as THAP9 [87]. THAP9 genes are found in human, primate, zebrafish, other vertebrates and Ciona genomes but are partially deleted and inactive in rodents [88–90]. Previous functional studies have shown that the human THAP9 protein is an enzymatically active transposase that can both excise and transpose *Drosophila* P elements in either *Drosophila* or human cells [91]. However, human THAP9 lacks the hallmarks of a mobile element, such as flanking terminal inverted repeats or the existence of multiple internally deleted copies [89]. Where in the human genome, the THAP9 protein binds and if it may cleave the genome at specific sites is under investigation.

**Figure 11.**
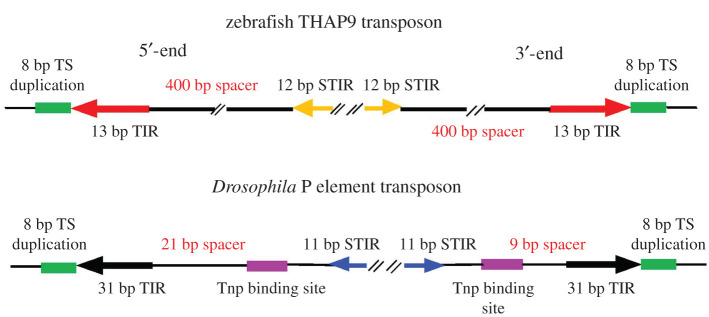
Comparison of P elements from zebrafish and *Drosophila*. (*a*) Zebrafish THAP9 transposon with 13 bp terminal inverted repeats (TIR) and 12 bp subterminal inverted repeats (STIR), with 400 bp spacers. (*b*) *Drosophila* P elements with 31 bp terminal inverted repeats (TIR) and 11 bp subterminal inverted repeats (STIR) with approximately 100 bp spacers. Both types of elements contain 8 bp direct target site duplications flanking the transposon insertions. Adapted from [50].

Interestingly, the zebrafish genome has one full-length THAP9 gene flanked by 13 bp terminal inverted repeats, 12 bp internal inverted repeats and 8 bp TSDs [89]. Zebrafish also have multiple copies of internally deleted P-like elements carrying the inverted repeats and 8 bp TSDs, suggesting that these elements were recently mobile (figure 11). Thus, P element-like transposons and THAP9 genes have expanded their presence among animal genomes outside of *Drosophila* and other insects.

## 8. Tissue-specificity of P element transposition: a paradigm of alternative pre-mRNA splicing regulation

The analysis of the full-length 2.9 kb P element indicated that four ORF could be linked by alternative splicing to encode two proteins: the full-length active 87 kDa transposase (by expression of all four ORF) and a truncated 66 kDa protein lacking the C-terminal one-third of the transposase, but carrying the N-terminal THAP DNA-binding domain. While only the 87 kDa product can catalyze P element transposition, the truncated transposase protein can bind to P-element DNA and act as a transpositional repressor [60,61]. Importantly, removal of the germline restriction and expression of active transposase in somatic cells could be brought about by genetically engineering a P element lacking the P element third intron (IVS3) [59]. Interestingly, expression of P element transposase in somatic tissues, which can be achieved using the stable P[Δ2-3] 99B transgene, caused pupal lethality when in the presence of 17 non-autonomous P elements from the Birmingham strain second chromosome (BIRM2) [92]. This stable source of active P element transposase has been used extensively in P element mutagenesis screens [93].

**Figure 12.**
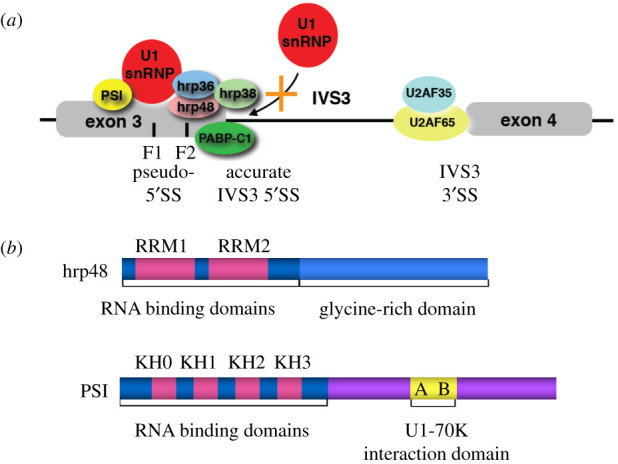
P element exonic splicing silencer complex and sequence-specific RNA-binding proteins. (*a*) The P element exonic splicing silencer complex (ESS) is assembled containing U1 snRNP and the RNA-binding proteins PSI, hrp48, hrp36, hrp38 and PABP-C. Assembly of this complex blocks U1 snRNP binding to the accurate IVS3 5′ splice site. (*b*) The RNA-binding proteins PSI and hrp48 bind specifically to the P element ESS. PSI has four N-terminal KH-type RNA-binding domains and a C-terminal region that interacts with U1-70 K protein. hrp48 contains two N-terminal RNP-CS type RNA-binding domains and a C-terminal RGG low complexity domain. Adapted from [50].

A series of both *in vitro* and *in vivo* experiments led to the identification of a negative RNA regulatory site upstream of IVS3 that interacted with RNA-binding proteins and U1 snRNP to inhibit IVS3 splicing [94–96]. Analysis of splicing reporter transgenes and *in vitro* biochemical studies indicated that a short splicing regulatory element, now called an exonic splicing silencer (ESS) element, is located upstream of the third intron (figure 12*a*) [94,96]. This sequence element, that when mutated can activate P element third intron splicing in somatic cells [94], contains two 5′ splice site-like sequences termed pseudo-5′ splice sites that can act as an ESS in *in vitro* splicing assays [95,96]. Using biochemical purification methods and *Drosophila* molecular genetics, two sequence-specific RNA-binding proteins, PSI (P element somatic inhibitor, a *Drosophila* counterpart of human FBP1, KSRP and FBP3) and hrp48 (a *Drosophila* counterpart of human hnRNPA1) were identified as functionally important for P element splicing repression (figure 12*b*) [97,98]. Biochemical analyses of the P element splicing silencer indicated that the ribonucleoprotein (RNP) complex that assembled on the silencer RNA contained U1 snRNP bound to one of the two pseudo-5′ splice sites [96,99]. The PSI protein, which has two protein repeat motifs A and B, can directly interact with the U1 snRNP 70 K protein [99], and it is believed to block the bound U1 snRNP from assembling active spliceosomes. In turn, the assembly of this inactive splicing silencer complex sterically blocks U1 snRNP from binding to the accurate IVS3 5′ splice site [96,99].

More recent studies using *in vitro* assembly of P element splicing silencer complexes on biotinylated RNA, affinity purification, mass spectrometry and splicing reporter assays identified three new proteins that functionally assemble on the P element silencer element: hrp36, hrp38 and cytoplasmic poly-A binding protein [100]. Most interestingly, the sequence-specific RNA-binding protein hrp48 that binds tightly to the P element silencer RNA [97] can recruit hrp36 and hrp38 to the P element silencer RNA through their C-terminal low complexity RGG domains [100]. The RGG domains, as well as other low complexity sequences on RNA-binding proteins, can lead to sequence-dependent condensates or liquid–liquid phase separation (LLPS), both *in vitro* and *in vivo* [101,102]. The specificity of LLPS/condensates often determines many aspects of protein–protein assembly in cells and in the nucleus.

## 9. A role for the piwi-interacting RNA (piRNA) and repressive histone marks in the control of transposon pre-mRNA splicing in the soma and germline

In somatic tissues, sequence-specific RNA-binding proteins PSI and hrp48 bind to the P element pre-mRNAs, blocking IVS3 intron splicing and serve to prevent fully spliced, and thereby full length, transposase expression [97,98]. However, PSI is not expressed in the female germline [98], and transposase expression in this tissue is thought to be primarily regulated by the piRNA pathway [33]. Typically, piRNAs control transposable element activity by inducing transcriptional silencing or post-transcriptional decay of mRNAs [34], ultimately leading to a decrease in the accumulation of target transcripts. However, expression analyses using FACS-sorted PGCs or adult ovaries show no or very limited changes in P element transcript accumulation in dysgenic germ cells when compared non-dysgenic controls, indicating that piRNAs do not primarily act to suppress P element transcript accumulation [33,48,103,104]. Instead, piRNAs were shown to regulate P element splicing by promoting the retention of third intron that is reminiscent of the regulation observed in somatic tissues [33,104]. Indeed, IVS3 splicing is only detected in the germline of dysgenic progeny or of mutants affecting the piRNA pathway [33,61]. Interestingly, even in this case, only a fraction of the P element transcripts seems to be fully spliced, suggesting that IVS3 splicing is either a generally inefficient process, likely to limit high levels of transposase expression [33,61]. Indeed, hrp48 is expressed in both the germline and soma [97]. Regardless, the qualitative effect of piRNAs on IVS3 splicing has been observed for endogenous P elements and recapitulated using reporter transgenes, with limited changes on transcript levels in both cases [33,48,104].

Despite being involved in the regulation of IVS3 splicing, the mechanisms by which somatically expressed RNA-binding proteins and germline-expressed piRNAs regulate intron retention seem to be mechanistically distinct. The somatically expressed RNA-binding protein, PSI, interacts specifically with the ESS element located just upstream of the third intron. By contrast, piRNA-mediated regulation relies on the binding of PIWI-piRNA complexes to target transcripts and evidence suggests that this process does not necessarily require the targeting to a specific sequence. Indeed, hybrid dysgenesis can be equally suppressed by *Drosophila* lines producing piRNAs throughout the entire element or against small P element segments distal to the IVS3 intron [30,104].

**Figure 13.**
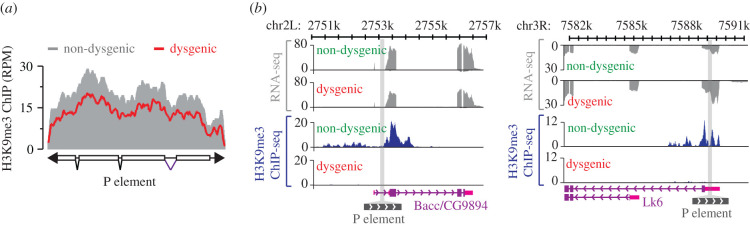
Loss of H3K9me3 histone marks over P element insertions during dysgenic crosses. (*a*) Normalized H3K9me3 ChIP signals over P elements in non-dysgenic (grey) and dysgenic (red) adult ovaries. (*b*) Genome browser view of two P element insertions showing transcriptional activity. Normalized RNA-seq and H3K9me3 ChIP signals are presented in grey and blue, respectively. The grey bar crossing the plots represents the P element chromosomal insertion site. Annotation is at bottom: purple boxes, coding exons; pink boxes, untranslated regions (UTR); purple lines, introns; grey box, P-element insertion. Views showing P-element insertion into the Bacc (also known as CG9894) (left) and Lk6 (right) genes. Adapted from [33].

Genetic analyses have revealed that IVS3 germline regulation is achieved indirectly by piRNA-mediated alterations of chromatin states. First, it relies on Piwi-interacting proteins such as Asterix/Gtsf1 and Panoramix/Silencio [33], which are dispensable for piRNA biogenesis but are essential for imposing chromatin changes on PIWI targets [34,105–108]. Second, P elements, as well as the genomic regions flanking transcriptionally active P element insertions, are enriched for the classic heterochromatic histone mark H3 lysine 9 trimethylation (H3K9me3) in non-dysgenic germ cells, compared to dysgenic progeny (figure 13) [33,48]. Indeed, Piwi-complexes are known to mediate the deposition of H3K9me3 [108]. Surprisingly, P element mRNA steady-state levels under dysgenic conditions or in piRNA pathway mutants display limited change despite the loss H3K9me3 [33,48]. This suggests that RNA polymerase II (Pol II) activity is not strongly influenced by the chromatin state of the P elements and that IVS3 alternative splicing may be regulated independently of changes in Pol II speed [109,110].

Interestingly, piRNA-mediated regulation of transposon alternative splicing does not seem to be exclusive to P elements, as a similar splicing regulation was also shown to modulate the expression of the Gypsy retrovirus-like element [33]. In this case however, the regulation is restricted to the ovarian somatic cells and Gypsy mRNA splicing favours the production of the Envelope protein mRNA [111], leading to the production of infectious particles that can spread into the surrounding tissues, most notably the germline [112,113].

## Data accessibility

This article has no additional data.
